# Supplementary material for: The effect of modifier and a water-soluble fertilizer on two forages grown in saline-alkaline soil
Source: PLoS One. 2024 Feb 29;19(2):e0299113. doi: 10.1371/journal.pone.0299113 (PMC10903894; doi:10.1371/journal.pone.0299113)
Supplement: S1 File — (DOCX) [file pone.0299113.s001.docx]

**The effect of modifier and a water-soluble fertilizer on two forages grown in saline-alkaline soil**

Shengchen Zhao^a^, Dapeng Wang^ꝉa^, Yunhui Li^b^, Wei Wang^b^, Jihong Wang^a^, Haibo Chang^a^*, Jingmin Yang^a^*

^a^College of Resource and Environmental Science, Jilin Agricultural University, Changchun 130118, Jilin Province, China.

^a^Key Laboratory of Saline-alkali Soil Improvement and Utilization, Ministry of agriculture and rural affairs

^b^College of Engineering, Jilin Normal University, Siping, Jilin Province, China

^ꝉ^This author contributed equally to this work and is co-first author of the paper.

E-mail: [changhb@jlau.edu.cn](mailto:changhb@jlau.edu.cn); [yangjingmin@jlau.edu.cn](mailto:yangjingmin@jlau.edu.cn)

S1 Table. The basic physical and chemical properties of saline-alkali soil

| Organic Matter g/kg | AvailableN  mg/kg | Available  P_2_O_5_  mg/kg | Available  K_2_O  mg/kg | pH  （1:2.5，20℃ water leaching） | alkalinity  % | Volume  Weight  g/cm^3^ | Total  Prosity  % | Na^+^  mg/kg | Ca^2+^ mg/kg | Mg^2+^ mg/kg |
| --- | --- | --- | --- | --- | --- | --- | --- | --- | --- | --- |
| 7.7 | 36.28 | 14.16 | 161.69 | 8.73 | 6.51 | 1.52 | 36.94 | 109.5 | 42.92 | 237.16 |

S2 Table. The nutrient content of the rice straw and the waste fungus chaff

| Experimental material | Organic Matter（%） | Total Nitrogen（%） | Total Phosphorus（%） | Total Potassium（%） |
| --- | --- | --- | --- | --- |
| Rice straw | 71.72 | 1.07 | 0.13 | 2.07 |
| Waste fungus chaff | 57.31 | 0.43 | 0.35 | 0.03 |

**Measuring indicators and methods**

After a period of 6 days from planting, the number of seedlings for both alfalfa and leymus chinensis was recorded. After 50 days, all the forage plants were removed from the soil, and the number of surviving plants was counted. The soil adhering to the root surface was carefully removed, and the forage plants were placed on a sieve, cleaned with water, and excess water was absorbed using filter paper. The forages from each treatment were stored separately and kept intact for further analysis.

Root dehydrogenase activity (DHA) was determined by dehydrogenase assay kit. The method was carried out according to the kit instructions, based on TTC colorimetric method[1]. The Content of malondialdehyde content (MDA) in roots was determined by malondialdehyde content determination kit, based on thiobarbituric acid colorimetry[2]. The total root length, total root surface area, average root diameter, and total root volume for the two kinds of forages were measured by the root scanning system.

The soil catalase activity (CAT) was determined by the kit with biochemical method. The specific steps were carried out in accordance with the instructions of the kit. An enzyme activity unit was defined by the degradation of 1μmol H_2_O_2_ per gram of soil sample per hour[3,4]. The activity of soil alkaline phosphatase (ALP) was measured using a kit with biochemical method. The specific measurement procedures were performed in accordance with the instructions provided with the kit. Based on the disodium phenyl phosphate colorimetric method[5], 1 nmol p-nitrophenol (PNP) was produced by hydrolysis of PNP per gram of soil sample per hour as an enzyme activity unit. Soil sucrase activity (SC) was determined by kit with biochemical method. The specific experiment steps were obtained according to the kit instructions. Based on the 3,5-dinitrosalicylic acid colorimetric method[5], 1 mg glucose was produced by per gram of soil sample in 24 h as an enzyme activity unit. Urease activity (URE) was determined by phenol sodium-sodium hypochlorite colorimetric method[5], expressed as the number of milligrams of NH_3_-N per gram of soil sample at 24 h.


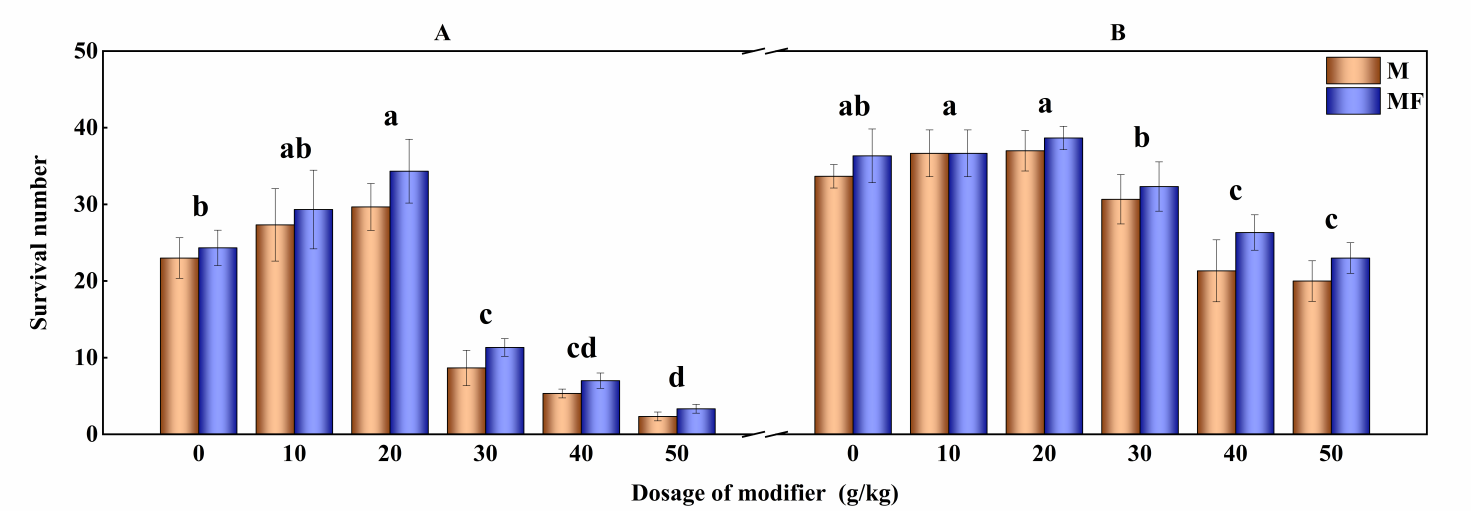


S1 Fig. Effects of modifier and water-soluble fertilizer on survival number of alfalfa.


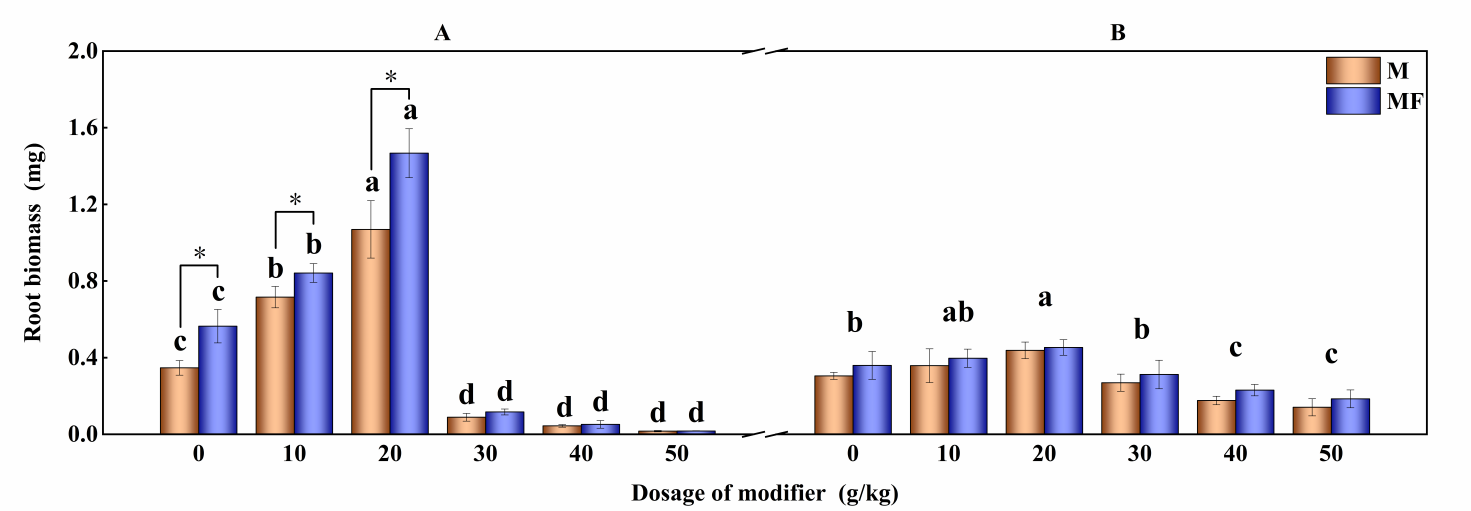


S2 Fig. Effects of modifier and water-soluble fertilizer on biomass in alfalfa roots.


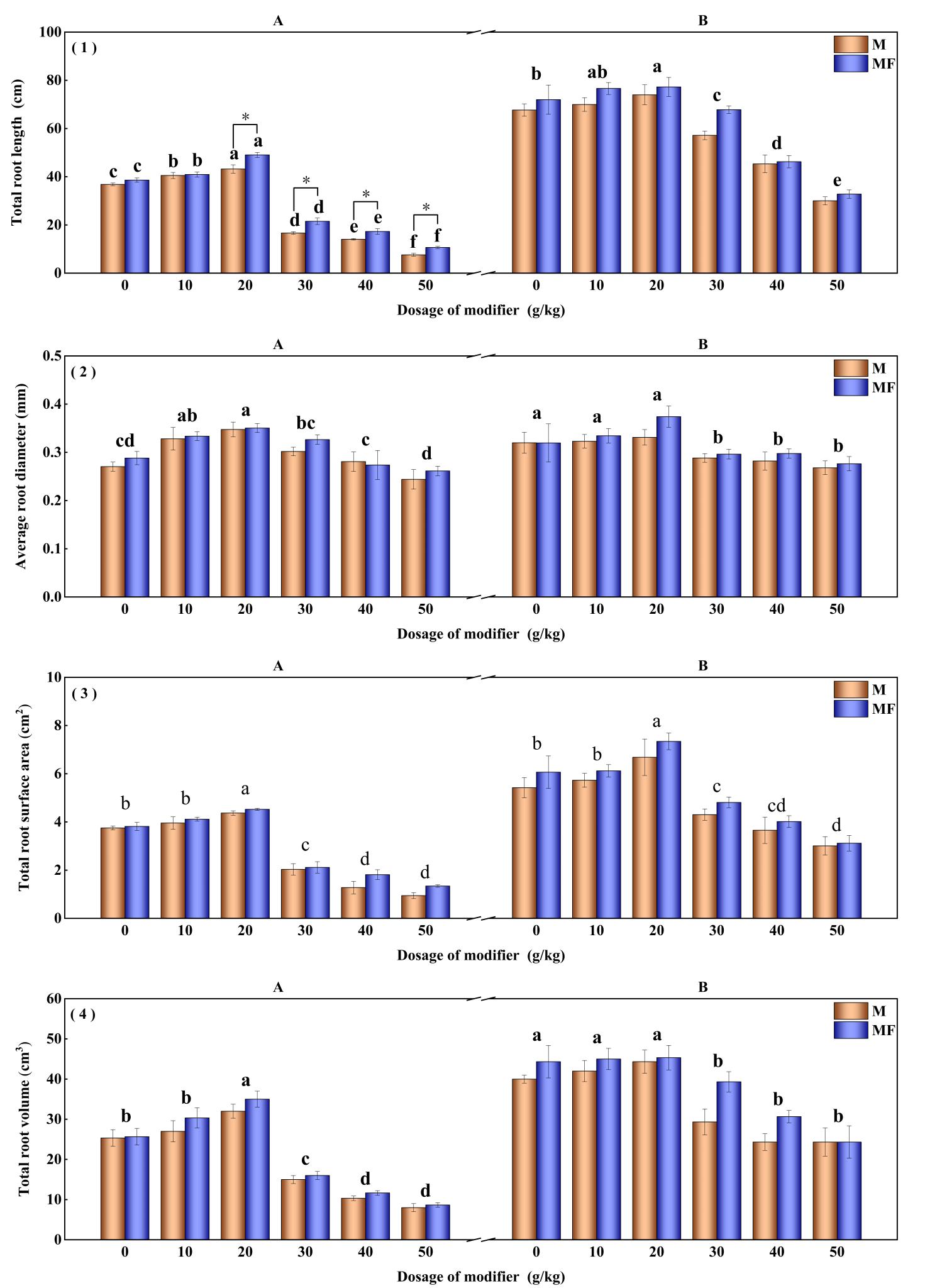


S3 Fig. Effects of modifier and water-soluble fertilizers on root growth of alfalfa.

(1) The root growth of alfalfa, (2) The root length of alfalfa,

(3) The root surface area of alfalfa and (4) The root volume of alfalfa.


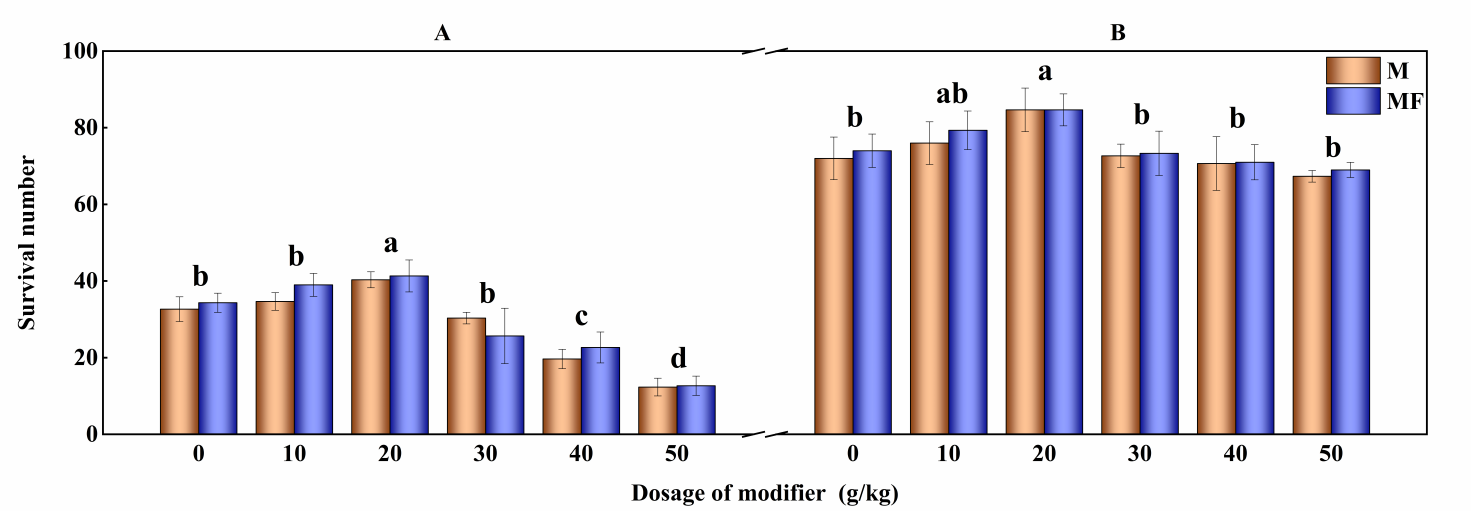


S4 Fig. Effects of modifier and water-soluble fertilizer on survival number of leymus chinensis.


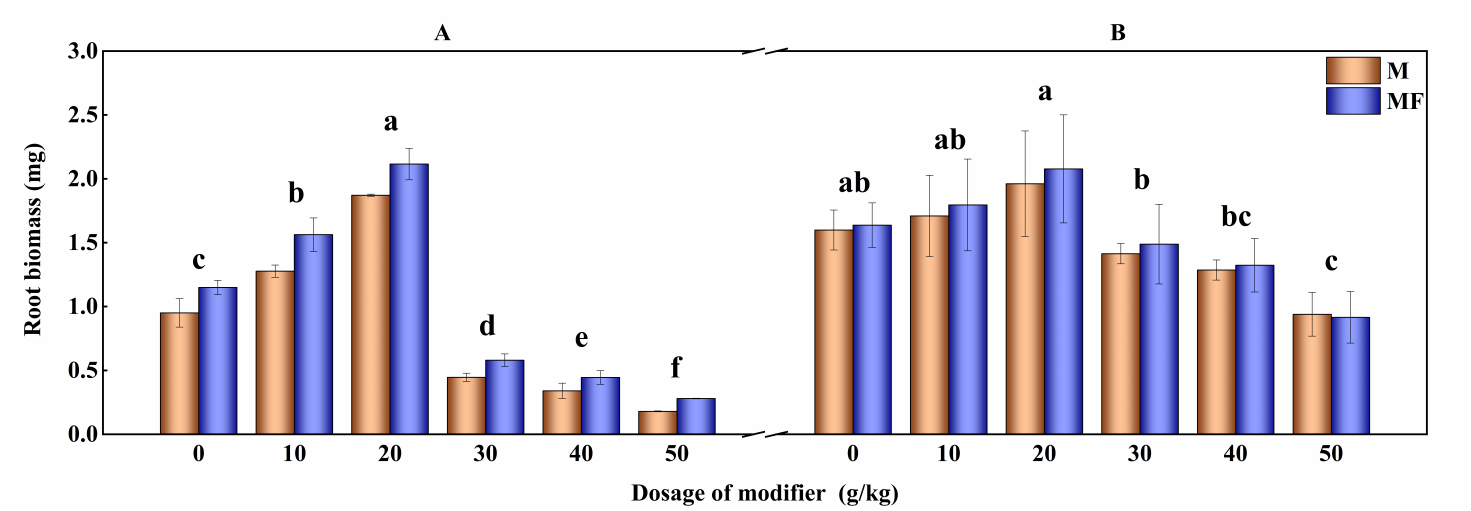


S5 Fig. Effect of modifier and water-soluble fertilizer on biomass in leymus chinensis roots.


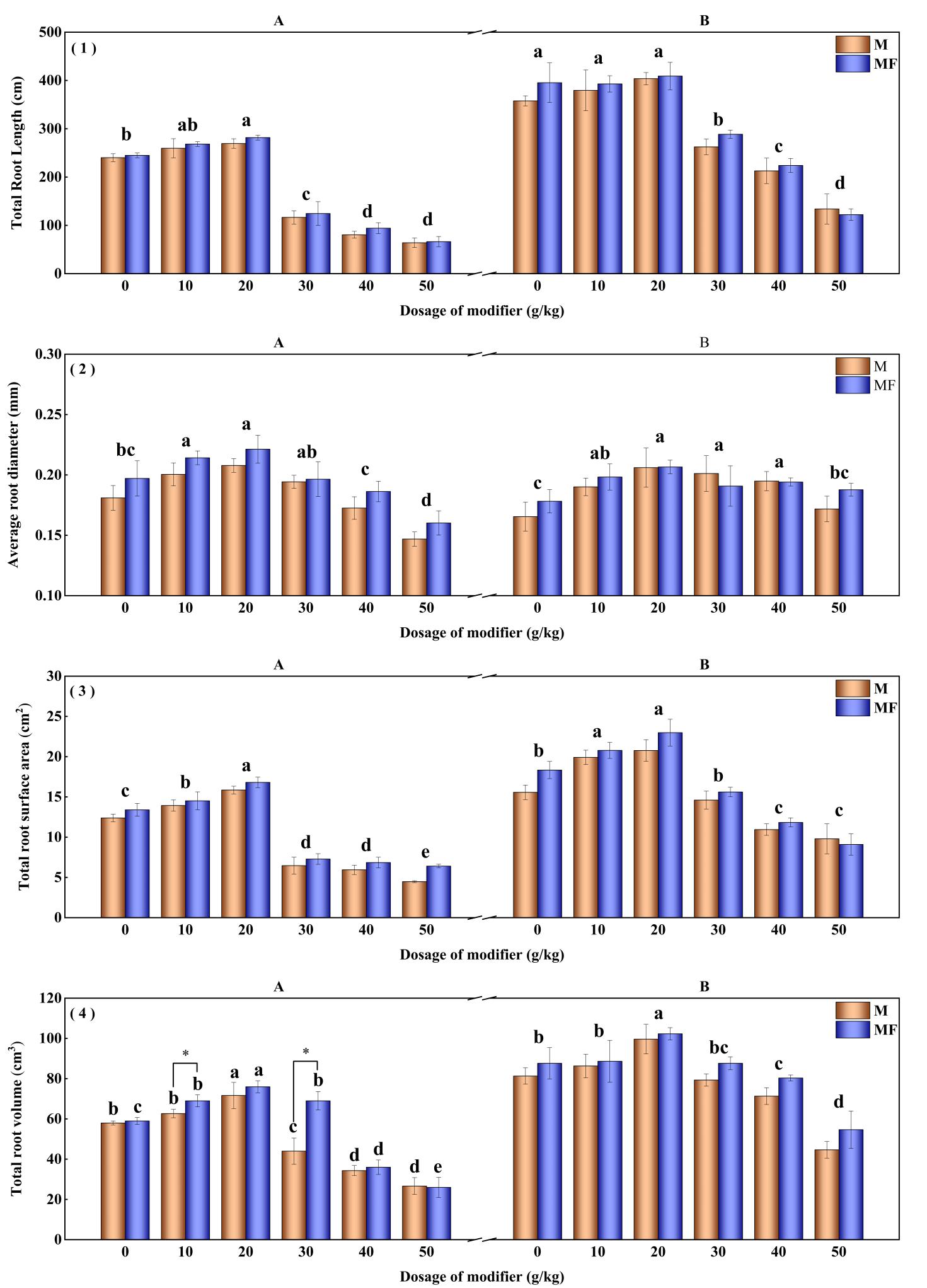


S6 Fig. Effects of modifier and water-soluble fertilizers on root growth of leymus chinensis.

(1) The root growth of leymus chinensis, (2) The root length of leymus chinensis,

(3) The root surface area of leymus chinensis and (4) The root volume of leymus chinensis.

References

1. Suzhou Keming biotechnology limited. Dehydrogenase kit instructions: TTC colorimetry[EB/OL]. http://www. cominbio. com.

2. Suzhou Keming biotechnology limited. Malondialdehyde content kit instructions: thiobarbituric acid[EB/OL]. http://www. cominbio. com.

3. Trasar-Cepeda, C., F. Camina, M. C. Leiros, and F. Gil-Sotres. 1999. "An improved method to measure catalase activity in soils." Soil Biology and Biochemistry 31(3): 483-485. https://doi.org/[10.1016/S0038-0717(98)00153-9](https://doi.org/10.1016/S0038-0717(98)00153-9)

4. Holz, F. 1987. "Antomatisierte photometrische Bestimmung der Aktivität von Bodenenzymen durch Anwendung (enzymatisch)-oxydativer Kupplungsreaktionen im Durchfluss. III. MItteilung: Die Bestimmung der beta-Glucosidaseaktivität." Landwirtschaftliche Forschung 39(4): 320-330.

5. Guan, S. Y. 1986. "Soil enzyme and its research methods." Beijing: Agriculture Press, 294-297.
