# Supplementary material for: The effect of modifier and a water-soluble fertilizer on two forages grown in saline-alkaline soil
Source: PLoS One. 2024 Feb 29;19(2):e0299113. doi: 10.1371/journal.pone.0299113 (PMC10903894; doi:10.1371/journal.pone.0299113)
Supplement: Attachment — Submitted filename: Response to Reviewers.docx [file pone.0299113.s003.docx]

Manuscript ID: PONE-D-23-28238

TITLE: The effect of modifier and a water-soluble fertilizer on two forages grown in saline-alkaline soil

Thank you very much for returning to us the Reviewers’ comments and queries. We also thank the Reviewers for their time and effort in evaluating our work. Please find our response to the points raised by the Reviewers in blue; changes in the manuscript are highlighted in red. We hope the revised manuscript can be accepted for publication on PLoS One.

Reviewer 2 comments:

1. Though most of the suggestions have been addressed but the introduction need further refinement with clear objectives and hypothesis.

Thanks for your suggestion. I have revised the Introduction in the manuscript.

Introduction revised in the manuscript:

Because of its porosity, biochar can improve soil physical structure[35], promote seed germination and crop growth[36], and improve soil enzyme activity and remove soil pollutants. Biochar itself is rich in carbon, and its ashes contains mineral elements needed for plant growth, which can improve soil permeability and increase soil carbon stock. In addition, the oxygen-containing functional groups on the surface of biochar, such as -COOH, -OH, etc., can react with the salt ions adsorbed in the soil colloid and adsorb Na^+^, which is the most representative of saline and alkaline soils, and thus improve the salinity and alkalinity of the soil, therefore, biochar has a broad application prospect in soil improvement[37, 38]. Application of water-soluble fertilizers improves soil quality and increases crop yields in alkaline soils[39].

37.	Hossain MZ, Bahar MM, Sarkar B, Donne SW, Ok YS, Bolan N. Biochar and its importance on nutrient dynamics in soil and plant. Biochar. 2020; 2: 379-420.

https://doi.org/[10.1007/s42773-020-00065-z](http://dx.doi.org/10.1007/s42773-020-00065-z).

38.	Yuan P, Wang J, Pan Y, Shen B. Review of biochar for the management of contaminated soil: Preparation, application and prospect. Science of the total environment. 2019; 659: 473-490.

[https://doi.org/10.1016/j.scitotenv.2018.12.400](https://doi.org/10.1016/j.scitotenv.2018.12.400).

39.	He K, Xu Y, He G, Zhao X, Wang C, Li S, Zhou G, Hu R. Combined application of acidic biochar and fertilizer synergistically enhances Miscanthus productivity in coastal saline-alkaline soil. Science of The Total Environment. 2023; 164811.

[https://doi.org/10.1016/j.scitotenv.2023.164811](https://doi.org/10.1016/j.scitotenv.2023.164811).

Best regards

Haibo Chang
