# Supplementary material for: The effect of modifier and a water-soluble fertilizer on two forages grown in saline-alkaline soil
Source: PLoS One. 2024 Feb 29;19(2):e0299113. doi: 10.1371/journal.pone.0299113 (PMC10903894; doi:10.1371/journal.pone.0299113)
Supplement: Attachment — Submitted filename: Response to Reviewers.docx [file pone.0299113.s004.docx]

Manuscript ID: PONE-D-23-28238

TITLE: The effect of modifier and a water-soluble fertilizer on two forages grown in saline-alkaline soil

Thank you very much for returning to us the Reviewers’ comments and queries. We also thank the Reviewers for their time and effort in evaluating our work. Please find our response to the points raised by the Reviewers in blue; changes in the manuscript are highlighted in red. We hope the revised manuscript can be accepted for publication on PLoS One.

Reviewer 2 comments:

1. Please replace soda saline-alkali soil with saline -alkali soil or sodic soil through out the manuscript. Because soda saline-alkali soils are same as sodic or saline -alkali soil. If author wish to explain it differently, please submit your response. The data in tabular form is minimal, as the table data give better idea of each treatments and their interaction with other factor. Hence can be included for better understanding.

Thanks for your suggestion. I have replaced soda saline-alkali soil with saline -alkali soil through out the manuscript. The basic properties of saline-alkali soil and The nutrient content of the rice straw and the waste fungus chaff The relevant data have been tabulated in the supporting information.

Introduction revised in the manuscript:

**Keywords:** saline-alkali soil, modifier, water-soluble fertilizers, alfalfa, leymus chinensis

The Songnen Plain with an area of 37,000 km^2^ is the largest saline-alkali land in China. Among the three major regions worldwide where salt and alkali are distributed, the Songnen Plain demonstrates the highest ecological degradation rate.

The saline-alkali soil sample was collected in Erlong Town, Taonan City, Jilin Province, which belonged to Songnen Plain.

Best regards

Haibo Chang
